# Potential effects of teriparatide (PTH (1–34)) on osteoarthritis: a systematic review

**DOI:** 10.1186/s13075-022-02981-w

**Published:** 2023-01-06

**Authors:** Guoqing Li, Su Liu, Huihui Xu, Yixiao Chen, Jiapeng Deng, Ao Xiong, Deli Wang, Jian Weng, Fei Yu, Liang Gao, Changhai Ding, Hui Zeng

**Affiliations:** 1 Department of Bone & Joint Surgery, Peking University Shenzhen Hospital, Shenzhen, 518036 People’s Republic of China; 2 National & Local Joint Engineering Research Center of Orthopaedic Biomaterials, Peking University Shenzhen Hospital, Shenzhen, 518036 People’s Republic of China; 3 Center for Clinical Medicine, Huatuo Institute of Medical Innovation (HTIMI), Berlin, Germany; 4 Sino Euro Orthopaedics Network (SEON), Berlin, Germany; 5 Clinical Research Centre, Zhujiang Hospital, Southern Medical University, Guangzhou, People’s Republic of China

**Keywords:** Teriparatide, Osteoarthritis, Systematic review, Treatment

## Abstract

Osteoarthritis (OA) is a common and prevalent degenerative joint disease characterized by degradation of the articular cartilage. However, none of disease-modifying OA drugs is approved currently. Teriparatide (PTH (1–34)) might stimulate chondrocyte proliferation and cartilage regeneration via some uncertain mechanisms. Relevant therapies of PTH (1–34) on OA with such effects have recently gained increasing interest, but have not become widespread practice. Thus, we launch this systematic review (SR) to update the latest evidence accordingly. A comprehensive literature search was conducted in PubMed, Web of Science, MEDLINE, the Cochrane Library, and Embase from their inception to February 2022. Studies investigating the effects of the PTH (1–34) on OA were obtained. The quality assessment and descriptive summary were made of all included studies. Overall, 307 records were identified, and 33 studies were included. In vivo studies (*n* = 22) concluded that PTH (1–34) slowed progression of OA by alleviating cartilage degeneration and aberrant remodeling of subchondral bone (SCB). Moreover, PTH (1–34) exhibited repair of cartilage and SCB, analgesic, and anti-inflammatory effects. In vitro studies (*n* = 11) concluded that PTH (1–34) was important for chondrocytes via increasing the proliferation and matrix synthesis but preventing apoptosis or hypertrophy. All included studies were assessed with low or unclear risk of bias in methodological quality. The SR demonstrated that PTH (1–34) could alleviate the progression of OA. Moreover, PTH (1–34) had beneficial effects on osteoporotic OA (OPOA) models, which might be a therapeutic option for OA and OPOA treatment.

**Supplementary Information:**

The online version contains supplementary material available at 10.1186/s13075-022-02981-w.

## Background

**Fig. 1:**
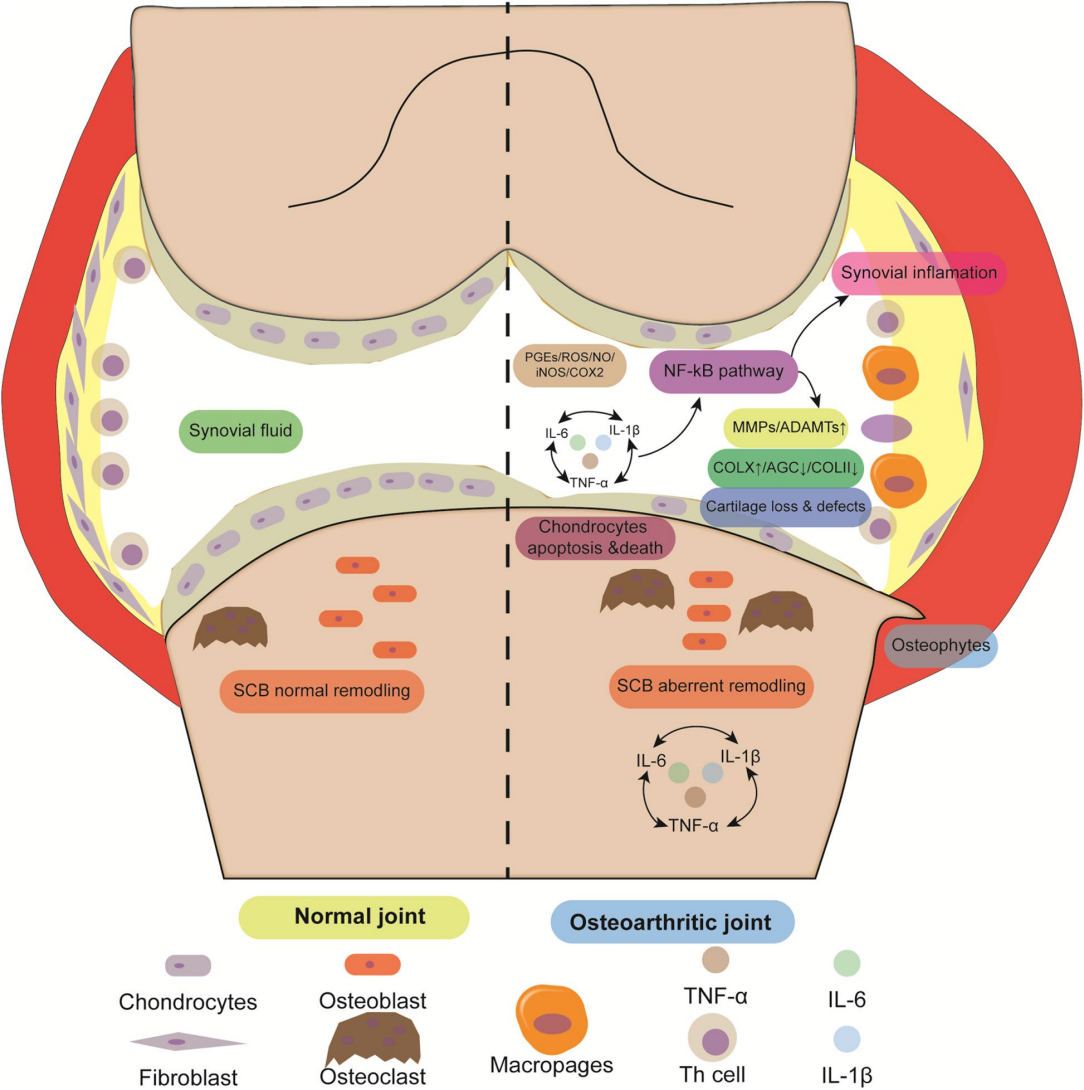
The schematic structures of normal (left part) and osteoarthritic (right part) joint. Multiple factors and pro-inflammatory cytokines resulting chondrocytes catabolism and ECM degradation in OA joints

Osteoarthritis (OA) is a common musculoskeletal disorder and prevalent degenerative disease worldwide [1, 2]. Both non-load bearing and load-bearing joints are affected by multiple factors such as trauma, senility, gender, genetics, and obesity [3], which resulted in functional disability or decreased quality of life. Articular cartilage is an avascular tissue, while chondrocytes are unique cellular components and responsible for the maintenance of the extracellular matrix (ECM) via the balance of catabolism and anabolism. Type II collagen (COL II) and aggrecan (AGC) are secreted proteins, which are essential for the integrity of cartilage. Break-down of chondrocytes is one of the molecular characteristics of OA, which is characterized by progressive damage including cartilage erosion, synovitis, and subchondral bone (SCB) disturbance. The normal metabolism of cartilage is disturbed by inflammatory cytokines such as tumor necrosis factor-α (TNF-α) and interleukin-1β (IL-1β), shifting to catabolism and ECM degradation [4]. Oxidative stress and apoptosis generate the decrease of chondrocytes and loss of cartilage [5]. The schematic diagram of normal and osteoarthritic joint was illustrated in Fig. 1.

Recommendation of OA treatment includes physiotherapy, pharmacological, and surgical interventions [6, 7]. Physiotherapy should be advocated due to its safety and effectiveness. However, limited choices and less effectiveness of drugs were restricted to symptom relief and accompanied by adverse effects [8]. Currently, no disease-modifying OA drugs (DMOADs) are available to alleviate the progression of OA. And therefore, strategies to protect the chondrocytes and the cartilage represent potential new therapeutic modalities.

Teriparatide (PTH (1–34)) contains 34 amino acids of parathyroid hormone, which was applied on the treatment of osteoporosis (OP) and bone fracture [9, 10] by maintaining calcium homeostasis, increasing cortical and trabecular thickness, and stimulating bone formation [11]. In addition, quantitative studies documented PTH (1–34) could mediate anabolic effects among chondrocytes [12] by enhancing chondral regeneration [13] and increasing ECM synthesis [14]. Experimental studies investigated the benefits of PTH (1–34) on OA pitiful without frequent practice or systematic review (SR). For these reasons, we reviewed the accessible research to update the effect of PTH (1–34) on OA.

## Methods

### Protocol

We performed this SR in accordance with the Preferred Reporting Items for Systematic Review and Meta-Analysis (PRISMA) statements [15]. We recorded the study protocol on the international Prospective Register of Systematic Reviews (PROSPERO) with code CRD42022315089.

### Literature search strategy

A comprehensive literature search was conducted in 5 databases (PubMed, Web of Science, Medline, the Cochrane Library, and Embase) from their inception to February 2022. The Medical Subject Headings (MeSH) terms and keywords were combined with boolean operators, “OR” or “AND”. The MeSH terms and keywords were as follows: “Teriparatide,” “hPTH (1–34),” “Human Parathyroid Hormone (1–34),” “Parathar,” “Teriparatide Acetate,” “Forteo,” “Osteoarthritis,” “Osteoarthritides,” “Osteoarthrosis,” “Osteoarthroses,” “Arthritis,” “Degenerative,” “Arthritides,” “Degenerative,” “Degenerative Arthritides,” “Degenerative Arthritis,” “Arthrosis,” “Arthroses,” “Osteoarthrosis Deformans.” In addition, the reference lists of all retrieved papers were further obtained manually. The search strategy of these five databases is provided in Additional file 1.

#### Inclusion and exclusion criteria

The eligible studies should meet the following criteria: (1) prospective and retrospective studies, randomized and controlled clinical trials; (2) patients or animal models with OA treated by PTH (1–34) directly or indirectly; and (3) studies published in the English language. Studies were excluded from this review if they were reviews, research protocols, abstracts only, commentaries, or editorials.

#### Study selection

All records of five databases were imported into the reference management software program Endnote X 9.3.3. After the removal of duplicates, two authors (GQL and SL) independently reviewed the titles and abstracts of the remaining records for relevance to the topic. Studies that potentially or completely met the inclusion criteria were kept and full texts were retrieved. The two authors (GQL and SL) independently assessed the full texts to decide whether to keep the records or not. A consensus meeting with a third reviewer (FY) was used to resolve discrepancies. The final included studies were reviewed by all authors for agreement.

#### Data extraction

The information of in vivo and in vitro studies was extracted in the standardized information forms: (1) first author’s surname, year of publication, and country; (2) subjects; (3) intervention; (4) dose and duration of treatment; (5) route; and (6) findings. Two investigators (GQL and SL) independently reviewed and extracted information from included studies. Disagreements were discussed with a third author (JW) to reach a consensus.

#### Quality assessment

The methodological quality of the in vivo studies was assessed by SYRCLE’s risk of bias tool [16] while the in vitro studies with Checklist for Reporting In-vitro Studies (CRIS) instruction [17]. Two authors (GQL and SL) independently assessed the methodological quality of the articles included, and discrepancies were resolved by discussion with a third author (FY).

## Results

### Identification of relevant studies

**Fig. 2:**
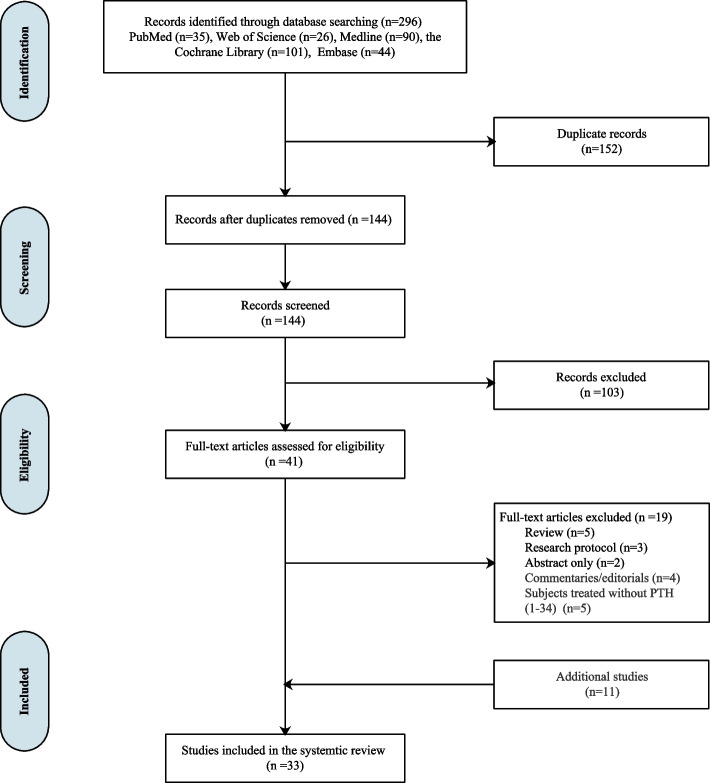
PRISMA flowchart (Preferred Reporting Items for Systematic Reviews). A comprehensive literature search was conducted and from their inception to February, 2022 and of 33 studies were included ultimately

The initial literature search resulted in 296 articles from PubMed (*n* = 35), Web of Science (*n* = 26), MEDLINE (*n* = 90), the Cochrane Library (*n* = 101), and Embase (*n* = 44) (Fig. 2). There are 152 duplicate records that were removed, and the remaining 144 records were screened by title and 103 records were excluded. Next, 41 full-text articles were assessed for their eligibility. Nineteen were excluded for (1) review (*n* = 5); (2) research protocol (*n* = 3); (3) abstract only (*n* = 2); (4) commentaries or editorials (*n* = 4); and (5) subjects treated without PTH (1–34) (*n* = 5). In addition, 11 additional records were added. Finally, 33 papers were considered and included.

### In vivo studies showed potential effects of PTH (1–34) on OA models

According to the inclusion criteria, 22 in vivo studies were included (Table 1). The studies were conducted in numerous countries including China (Shao et al., 2020 [18]; Shao et al., 2021 [19]; Chen et al. 2021 [20]; Chen et al. 2018 [21]; Rajalakshmanan et al. 2012 [22]; Ma et al. 2017 [23]; Zhang et al. 2022 [24]; Chang et al. 2009 [25]; Yan et al. 2014 [26]; Dai et al. 2016 [27]; Cui et al. 2019 [28]; He et al. 2021 [29];  Longo et al. 2020 [30]), Germany (Orth et al. 2014 [31]; Orth et al. 2013 [32]), and the USA (Dutra et al. 2017 [33]; Sampson et al. 2011 [34]; Brien et al. 2017 [35]; Bagi et al. 2015 [36]; Antunes et al. 2013 [37]), Spain (Lugo et al. 2012 [38]; Bellido et al. 2011 [39]).

**Table 1:** Characteristics of in vivo studies about the effect of teriparatide on OA

Author (year, country)	Subjects	Intervention	Dose (duration)	Route	Findings
Shao et al. (2020, China) [18]	CIOA mouse	PTH (1–34)	10/40 μg/kg (6 weeks)	SC	PTH (1–34) exhibits protective effects on both cartilage and SCB in a dose-dependent manner via the JAK2/STAT3 signaling pathway
Shao et al. (2021, China) [19]	CIOA mouse	PTH (1–34)	40 μg/kg (6 weeks)	SC	PTH (1–34) exhibits protective effects on both cartilage and SCB by down-regulating the expression of JAK2/STAT3 and WNT5A/ROR2
Chen et al. (2021, China) [20]	Guinea pig	PTH (1–34)	10 nM (12 weeks)	IA	PTH (1–34) improves spontaneous OA by directly affecting the cartilage rather than the SCB or metaphyseal bone
Chen et al. (2018, China) [21]	ACLT Rats	PTH (1–34)	10 nM (5 weeks)	IA	PTH (1–34) alleviates OA progression after ACLT and histological molecular changes by reducing chondrocyte terminal differentiation and apoptosis and by increasing autophagy
Eswaramoorthy et al. (2012, China) [22]	PIOA Rat	PTH (1–34)	0.4 mg (5 weeks)	IA	PTH (1–34) has beneficial effects on suppressing early OA progress PLGA microsphere-encapsulated PTH (1–34) with a controlled-release property represents a potent method to treat early OA
Ma et al. (2017, China) [23]	SD rats	PTH (1–34)	15 μg/kg (2/6 weeks)	SC	PTH (1–34) up-regulates the Wnt/β-catenin signaling pathway and down-regulated RUNX2 through an alternative pathway
Zhang et al. (2022, China) [24]	Patellar ligament shortening SD rats	PTH (1–34)	30 μg/kg (10 weeks)	SC	PTH (1–34) could improve cartilage metabolism and SCB health in early PFJOA model
Chang et al. (2009, China) [25]	CIOA Rats	PTH (1–34)	10 nM (10 days)	SC	PTH (1–34) treats early OA without affecting normal chondrocytes, which might a potential effectiveness of the agent for OA treatment
Yan et al. (2014, China) [26]	Guinea pigs	PTH (1–34)	15 μg/kg (3/6 months)	SC	PTH (1–34) prevents cartilage damage progression and retard the deterioration of SCB
Dai et al. (2016, China) [27]	Guinea pigs	PTH (1–34)	24 μg/kg (12 weeks)	SC	Both celecoxib and PTH (1–34) exhibit protective effects on cartilage degeneration in menisc-ectomized guinea pigs PTH (1–34) exhibits superior performance to celecoxib not only in metabolism of cartilage tissue but also in maintenance of SCB micro-architecture
Cui et al. (2019, China) [28]	C57BL/6 J	PTH (1–34)	40 μg/kg (4 weeks)	SC	PTH (1–34) reduces the accumulation of senescent cells in SCB by inhibiting p16 and improves bone marrow microenvironment to active bone remodeling process, indicating a potential preventative and therapeutic treatment for age-related OA
He et al. (2021, China) [29]	DMM OA mice	PTH (1–34)	80 μg/kg (4 weeks)	SC	PTH (1–34) has an obvious analgesic and anti-inflammatory effect, inhibits the matrix synthesis, and alleviates the OA progression PTH (1–34) inhibited TNF-α expression and antagonized TNF-α-induced MMP13 expression via the PKA pathway and the NF-κB signaling pathways
Longo et al. (2020, China) [30]	Meniscectomy Dogs	PTH (1–34)	2.4 μg/kg (3 weeks)	IA	PTH (1–34) promotes the regenerative and chondroprotective effects of the tissue-engineered meniscus total implantation in a canine model by inhibiting the terminal differentiation of BMSC chondrogenesis and degeneration of knee joint cartilage
Orth et al. (2014, Germany) [31]	Rabbits	PTH (1–34)	10 mg/kg (6 weeks)	SC	PTH (1–34) causes broadening of the calcified cartilage layer and resulting in osteoarthritic cartilage degeneration PTH (1–34)-induced alterations of the normal SCB microarchitecture may provoke early OA
Orth et al. (2013, Germany) [32]	Rabbits osteochondral defects	PTH (1–34)	10 μg/kg (6 weeks)	SC	PTH (1–34) stimulates articular cartilage and SCB repair, which emerges as a promising agent in the treatment of focal osteochondral defects
Dutra et al. (2017, USA) [33]	C57BL/6 J	PTH (1–34)	80 μg/kg (21 days)	SC	PTH (1–34) results in early mineralization of the MCC and cartilage degeneration PTH (1–34) induces alteration in the microarchitecture of the MCC and the SCB
Sampson et al. (2011, USA) [34]	MLI OA mice	PTH (1–34)	40 μg/kg (8 weeks)	SC	PTH (1–34) may be useful for decelerating cartilage degeneration and inducing matrix regeneration in OA model
O'Brien et al. (2017, USA) [35]	Transgenic mice	PTH (1–34)	80 μg/kg (2 weeks)	SC	PTH (1–34) increases the number of Col1a1/Col2a1/Col10a1-positive cells; bone volume fraction, tissue density and trabecular thickness of the SCB; proteoglycan distribution with a concomitant increase in MCC mineralization; chondrocytes differentiation and increases mineralization
Bagi et al. (2015, USA) [36]	Posttraumatic OA Rats	PTH (1–34)	40 μg/kg (10 weeks)	SC	A single drug will have the capacity to reduce joint inflammation, curb excessive bone remodeling, improve cartilage regeneration, and reduce pain Both Zol and PTH does not prevent or correct the deterioration of the hyaline cartilage, thickening of the SCB plate, osteophyte formation, and mechanical incapacity of the OA
Antunes et al. (2013, USA) [37]	Prg4 mutant mice	PTH (1–34)	50 μg/kg (6 weeks)	SC	SCB contributes to the disruption of the articular cartilage in Prg4 mutant mice PTH (1–34) could not demonstrate a protective effect in the arthropathic joints because of Prg4 mutant
Lugo et al. (2012, Spain) [38]	OVX and ACLT rabbits	PTH (1–34)	10 mg/kg (10 weeks)	SC	PTH (1–34) ameliorates OA by improving SCB integrity, inhibiting cartilage degradation, and exerting certain beneficial effects on synovial changes PTH (1–34) exhibits direct beneficial effects upon the synovium of this experimental model PTH (1–34) administration might hold a potential as therapeutic option for synoviopathy associated with OA
Bellido et al. (2011, Spain) [39]	OVX and ACLT rabbits	PTH (1–34)	10 μg/kg (10 weeks)	SC	PTH (1–34) prevents cartilage damage progression and microstructural and remodeling of SCB in rabbits with early OPOA

*OA* Osteoarthritis, *CIOA* Collagenase-induced osteoarthritis, *PTH (1*–*34)* Teriparatide, *SC* Subcutaneous injection, *SCB* Subchondral bone, *IA* Intra-articular, *ACLT* Anterior cruciate ligament transection, *MCC* Mandibular condylar cartilage, *PLGA* Poly lactic-co-glycolic acid, *PIOA* Induced osteoarthritis, *OVX* Ovariectomized, *SD* Sprague–Dawley, *PFJOA* Patellofemoral joint osteoarthritis, *nM* nmol/L, *MLI* Meniscal ligamentous injury, *OPOA* Osteoarthritis preceded by osteoporosis, *DMM* Destabilization of the medial meniscus

Studies implied that PTH (1–34) exhibited protective effects on both cartilage and SCB. Shao et al. concluded similar findings among collagenase-induced OA (CIOA) mouse models in a dose-dependent manner via the JAK2/STAT3 and WNT5A/ROR2 signaling pathway [18, 40]. At the dose of 10 μg/kg/day of PTH (1–34), Orth et al. reported that PTH (1–34) could broaden the calcified cartilage layer, result in cartilage degeneration, and induce alterations in the microarchitecture of SCB to provoke early OA [31]. Moreover, PTH (1–34) would stimulate articular cartilage and SCB repair [41]. Bellido et al. suggested that PTH (1–34) could improve microstructural and remodeling parameters of SCB, which contributed to preventing cartilage damage and OA progression in OVX and ACLT rabbits [39].

PTH (1–34) would reduce the predisposing factors for OA progression. At the dose of 40 μg/kg/day, Cui et al. believed that PTH (1–34) reduced the accumulation of senescent cells in SCB by inhibiting p16 for age-related OA [28]. In addition, Sampson et al. considered that it might be useful to decelerate cartilage degeneration among meniscal ligamentous injury (MLI) mice and induce ECM regeneration among OA patients [34]. Bagi et al. concluded that PTH (1–34) would reduce joint inflammation, curb excessive bone remodeling, improve cartilage regeneration, and reduce pain in post-traumatic OA rats [36]. At the dose of 80 μg/kg/day of PTH (1–34), Dutra et al. found that it could result in mineralization and alteration of the mandibular condylar cartilage (MCC), with cartilage degeneration and abnormal remodeling of the SCB [33]. He et al. concluded that PTH (1–34) had an obvious analgesic and anti-inflammatory effect on DMM mice via the PKA and the NF-κB signaling pathways [29]. Brien et al. concluded that it would increase the differentiation and mineralization of chondrocytes as well as density of the SCB among the transgenic mice [35].

PTH (1–34) prevents cartilage damage and retards the deterioration of SCB. Yan et al. concluded that 15 μg/kg/day of PTH (1–34) protected the cartilage among guinea pigs [26]. Dai et al. found that 24 μg/kg/day of PTH (1–34) exhibited protective effects on cartilage degeneration among meniscectomy guinea pigs, which exhibited superior performance to celecoxib in both cartilage metabolism and maintenance of SCB micro-architecture [27]. Antunes et al. argued that SCB contributed to the disruption of the cartilage, but PTH (1–34) protected the destruction of the SCB [37]. Zhang et al. supposed that PTH (1–34) improved cartilage metabolism and SCB health on patellar ligament shortening SD rats [42].

Different routines would differ the effect of PTH (1–34). Eswaramoorthy et al. found that controlled-release property of PTH (1–34) via intra-articular (IA) injection suppressed early stages of OA in papain-induced OA (PIOA) rats [22]. Chen et al. suggested that PTH (1–34) improved spontaneous OA by directly affecting the cartilage rather than the SCB or metaphyseal bone [43], reduce chondrocyte terminal differentiation and apoptosis, and increase autophagy on ACLT rats via IA injection [44]. Longo et al. concluded that PTH (1–34) promoted the regenerative and chondroprotective effects of the tissue-engineered meniscus by inhibiting the differentiation of mesenchymal stem cells (BMSC) chondrogenesis and cartilage degeneration among the meniscectomy dogs [30], which represented a promising method to increase the chance of regeneration in the tissue-engineered meniscus.

### In vitro studies showed potential mechanism of PTH (1–34) intracellularly

Based on the inclusion criteria, 11 in vitro investigations were included in the SR (Table 2). These studies were conducted in numerous countries including China (Chang et al. 2009 [25], Shao et al. 2022 [45]; Chang et al. 2016 [46]), Canada (Mwale et al. 2010 [47]), USA (Funk et al. 1998 [48]), Sweden (Petersson et al. 2006 [49]), Australia (Music et al. 2020 [50]), Japan (Tsukazaki et al. 1996 [51]; Dogaki et al. 2016 [52]; Hosokawa et al. 2015 [53]), and Netherlands (Rutgers et al. 2019 [54]).

**Table 2:** Characteristics of in vitro studies about the effect of teriparatide on OA

Author (year, country)	Subjects	Intervention	Dose (duration)	Route	Findings
Chang et al. (2009, China) [25]	Human articular chondrocytes	PTH (1–34)	10 nM (10 days)	Co-culture	PTH (1–34) reverses the progression of terminal differentiation of human articular chondrocytes PTH (1–34) could be used to treat early OA without affecting normal chondrocytes
Shao et al. (2022, China) [45]	BMSCs	PTH (1–34)	10 nM (48 h)	Co-culture	PTH (1–34) alleviates OA by increasing the migration, proliferation, and chondral matrix formation of OA chondrocytes by inhibiting proinflammatory cytokines
Chang et al. (2016, China) [46]	Human articular chondrocyte	PTHrP	10^−8^ to 10^−7^ M (7 days)	Co-culture	PTH (1–34) is beneficial for preventing the chondro-degenerative changes initiated by dexamethasone treatment
Mwale et al. (2010, Canada) [47]	Human MSCs	PTH (1–34)	100 nM (48 h)	Co-culture	p38 and AKT protein kinase signaling pathways may not be required to initiate the regulation of expression of COLII and COLX by PTH (1–34), which is necessary for preventing precocious MSC hypertrophy
Funk et al. (1998, USA) [48]	RA and OA synovial tissue	PTHrP (1–40)/PTHrP (60–72)/PTHrP (1–86)	0.3 pM (24 h)	Co-culture	Proinflammatory cytokine-stimulated production of NH2 terminal PTHrP by synovial tissue directly invading cartilage and bone in RA, which might mediate joint destruction through direct effects on cartilage or indirectly via the induction of mediators of bone resorption
Petersson et al. (2006, Sweden) [49]	RA or OA Chondrocytes	PTHrP (1–34)	0.1 to 100 nM (15 days)	Co-culture	PTHrP (1–34) increases proliferation of human chondrocytes PTHrP (1–34) increases the amount of YKL-40 from chondrocytes derived from RA patients
Music et al. (2020, Australia) [50]	BMSCs	PTH (1–34)	0, 1, 10, or 100 nM (14 days)	Co-culture	PTH (1–34) suppresses BMSC hypertrophic gene expression in chondrogenic cultures PTH (1–34) has an anti-hypertrophic effect and a catabolic effect on BMSC as they become increasingly differentiated
Tsukazaki et al. (1996, Japan) [51]	Human chondrocytes	PTH (l–34)/hPTHrP (l–141)/hPTHrP (100–114)	10^−13^ to 10^−7^ M (120 min)	Co-culture	PTHrP is thought to be an important autocrine/paracrine factor for chondrocyte metabolism No significant difference of exogenously PTHrP (1–141) regard to the action of these agents, cell growth, differentiation
Dogaki et al. (2016, Japan) [52]	Hematoma-derived progenitor cells	PTH (1–34)	100 nM (14 days)	Co-culture	Pulsatile PTH (1–34) works on human cartilages in regarding to proliferation, osteogenic, and chondrogenic differentiation PTH (1–34) administration after fracture might positively act on other cells that contribute to fracture healing
Hosokawa et al. (2015, Japan) [53]	ATDC5 cells	PTH (1–34)	10^−10^/10^−9^/10^−8^ M (21 days)	Co-culture	PTH (1–34) regulates ATDC5 cells in both chondrogenesis and the circadian clock as time-dependent properties of chondrocyte function and differentiation
Rutgers et al. (2019, Netherlands) [54]	Human chondrocytes	PTH (1–34)	0.1 or 1.0 μM (4 weeks)	Co-culture	PTH (1–34) inhibits healthy human articular chondrocytes regeneration other than hypertrophic differentiation PTH (1–34) may be suitable for cartilage repair based on MSCs

*OA* Osteoarthritis, *BMSCs* Bone marrow mesenchymal stem cells, *PTH (1–34)* Teriparatide, *nM* nmol/L, *PTHrP* Parathyroid hormone-related protein, *M* mol/L, *MSC* Mesenchymal stem cells, *COL II* Type II collagen, *COLX* Type X collagen, *RA* Rheumatoid arthritis, *pM* pmol/L, *μM* μmol/L

As for the effects on human articular chondrocytes, PTH (1–34) influenced its differentiation and regeneration. Tsukazaki et al. concluded that PTHrP was an important autocrine and paracrine factor for chondrocyte metabolism as for cell growth and differentiation [51]. Rutgers et al. suggested that PTH (l–34) inhibited healthy human articular chondrocyte regeneration but did not influence hypertrophic differentiation [54]. Chang et al. concluded that PTH (l–34) could reverse the terminal differentiation of chondrocytes without affecting normal chondrocytes, while PTHrP prevented the chondrocyte degeneration initiated by dexamethasone [25]. Moreover, Chang et al. held that PTH (1–34) treated early OA without affecting normal chondrocytes [55]. When PTH (1–34) was applied for RA or OA chondrocytes treatment, the survival and inflammatory cytokines would be affected. Petersson et al. found that PTH (1–34) increased the proliferation of chondrocytes from human and RA patients [49]. However, Funk et al. revealed that the PTHrP could be examined in synovium and synoviocytes obtained from RA patients, which help to clarify the pathogenesis of RA to a certain extent and remain to be investigated further [48]. In addition, Lugo et al. found that PTH (1–34) ameliorated OA by improving SCB integrity, inhibiting cartilage degradation, and exerting effects on synovial changes [38]. PTH (1–34) held potential therapeutic option for synoviopathy associated with OA.

PTH (1–34) protected MSC with various effects. Shao et al. maintained that PTH (1–34) worked on MSC by increasing the migration, proliferation, ECM formation, and inhibiting proinflammatory cytokines [56]. Mwale et al. argued that PTH (1–34) helped to prevent precocious MSC hypertrophy [47]. Music et al. believed that PTH (1–34) suppressed MSC hypertrophic [50]. Dogaki et al. implied that PTH (1–34) may not have a positive effect at the fracture site because no positive effect was noticed when the fracture haematoma-derived progenitor cells were treated with PTH (1–34) [52]. Hosokawa et al. indicated that PTH (1–34) could reset the circadian rhythm of ATDC5 cells, which is expected to be useful to assess the molecular mechanisms of PTH (1–34) on chondrogenic differentiation [53]. PTH (1–34) played a significant role in chondrocytes through affecting the proliferation and ECM synthesis.

### Quality assessment of included studies

**Fig. 3:**
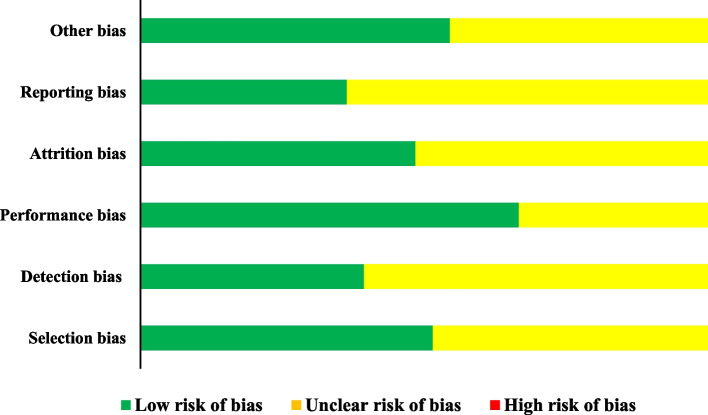
Risk of bias assessment across the studies (*n* = 33). Methodological quality was assessed for all involved studies

Methodological quality was assessed for all 33 involved studies (Fig. 3). An unclear risk of selection bias (because of lacking data regarding randomization method: *n* = 16); detection bias (blinding of outcome assessment, *n* = 20); performance bias (because of absent data about blinding of subjects, *n* = 11), attrition bias (*n* = 17), reporting bias (*n* = 21), and other bias (*n* = 15) were found.

## Discussion

To our knowledge, this is the first SR evaluating the existing papers about the effect of PTH (1–34) on OA regarding in vivo and in vitro investigations. The chondro-protective and cartilage-regenerative effects were reviewed, indicating that PTH (1–34) might be a potential preventative and therapeutic treatment for OA.

OA is the most prevalent degenerative joint disease with complicated pathogenesis characterized with damage to cartilage, narrow synovial cavity, invasion of the SCB, formation of osteophytes, and synovitis [57]. OP is a metabolic bone disease with decreased bone strength but increased fracture risk. OP and OA are common clinical conditions with high prevalence among older adults. Antiresorptive agents exhibited effects on bone mineralization and cartilage degradation for OA or OPOA [58]. However, treatments with polypharmacy for OA are limited to pain relief with less effective, which should be individualized to reduce the risk of side effects [59]. And therefore, DMOADs are highly demanded for OA or OPOA.

Quantitative studies indicated that PTH (1–34) played a significant role in calcium metabolism with an anabolic effect in the treatment of OP, fracture healing, non-union and stress fracture, augmentation of implant fixation, and chondro-protection in OA [14, 60]. In addition, PTH (1–34) could be a systemic pharmacology for OA by influencing cartilage quality such as ECM and chondrocyte contents [61]. The effects of PTH (1–34) were involved in decreasing COLX or RUNX2 but increasing AGC [34], which not only inhibited matrix metallopeptidase 13 (MMP13) or ADAM metallopeptidase with thrombospondin type 1 motif 4 (ADAMTS4), but also enhanced COLII and AGC [26, 42]. Moreover, PTH (1–34) reversed terminal differentiation towards hypertrophy and decreased apoptosis of chondrocytes [46, 47].

**Fig. 4:**
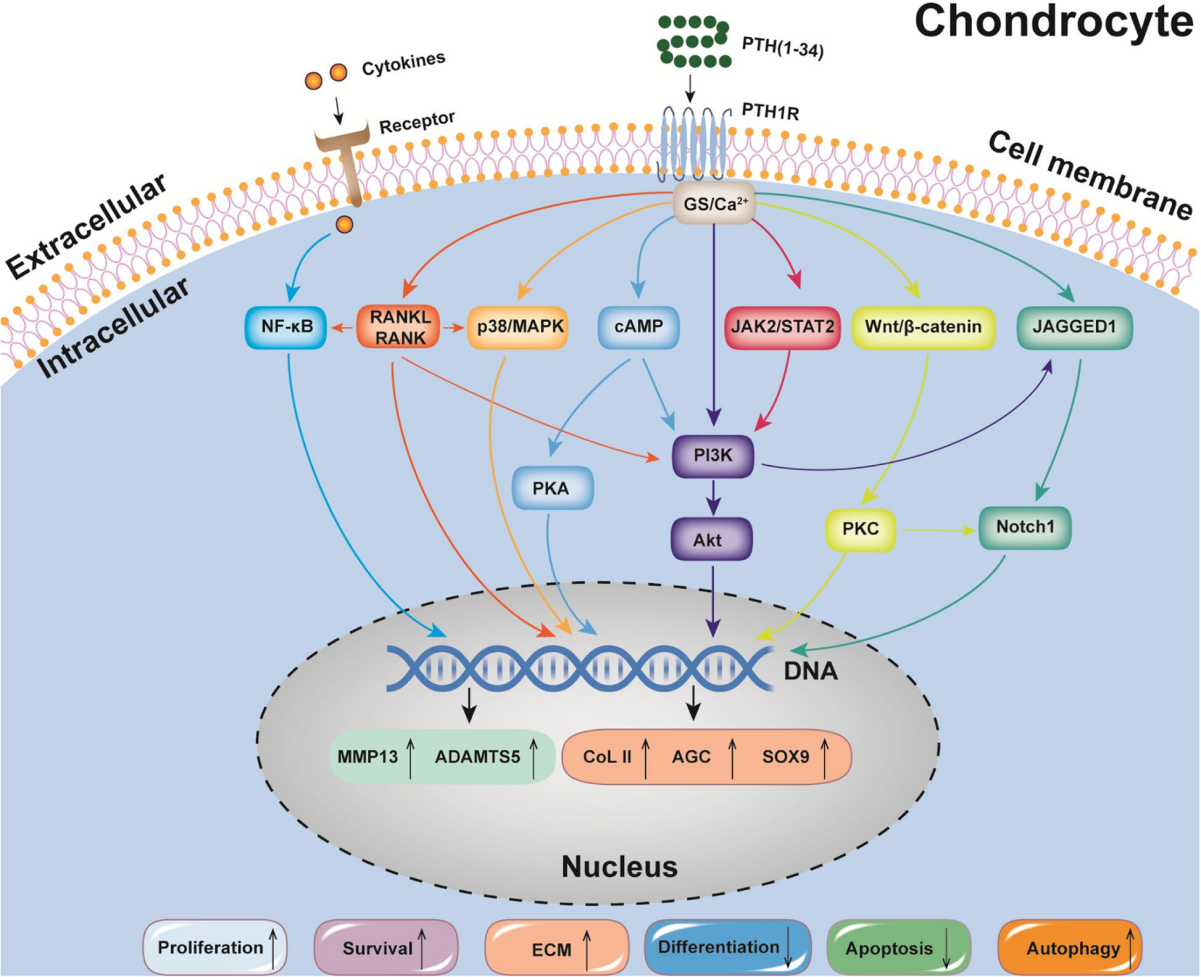
Relevant mechanisms of PTH (1–34) in the chondrocytes. The chondrocyte-protective and chondrocyte-regenerative effect of PTH (1–34) were explained by multiple mechanisms

The anabolic effects of PTH (1–34) on both cartilage and SCB were explained by multiple mechanisms (Fig. 4). The activation of NF-κB elevated inflammatory mediators of IL-1β, TNF-α, cyclooxygenase-2 (COX2), and inducible nitric oxide synthase (iNOS), which resulted in the initiation of OA and regulated the levels of MMP13 [62]. It is well established that the parathyroid 1 receptor (PTH1R) was a key regulator to induce differentiation and endochondral ossification by inducing ECM synthesis, suppressing maturation, and inhibiting degeneration [20]. PTH (1–34) elevated the expression of PTH1R, osteoprotegerin (OPG), and receptor activator of NF-κB ligand (RANKL) via the OPG/RANKL/RANK signaling pathway [26]. The Notch pathway was activated by PTH (1–34) with increased expression of JAGGED1 [63]. The expression of TNF-α was inhabited by PTH (1–34) via the PKA signaling pathway [29]. PTH (1–34) inhibited chondrocyte differentiation towards hypertrophy via the p38 and the p-AKT signaling pathway [47]. PTH (1–34) downregulated JAK2/STAT3 and Wnt5A/ROR2 [19] but upregulated the Wnt/β-catenin through an alternative signaling pathway [64].

In addition, the attenuation of signaling pathways including oxidative stress and apoptosis had an indispensable role in OA. Autophagy was a protective mechanism in normal cartilage. PTH (1–34) alleviated OA progression by reducing terminal differentiation, reducing apoptosis, and increasing autophagy via the mechanistic target of rapamycin (mTOR) and p62 [21]. Apoptosis was reversed, while both Bcl-2 and Bax were upregulated by PTH (1–34). Moreover, PTH (1–34) might reduce the accumulation of senescent cells by inhibiting p16 [28]. Both the sustained and intermittent action of PTH (1–34) suppressed OA effectively [22, 65]. IA application would directly affect the cartilage rather than the SCB or metaphyseal bone [43]. PTH (1–34) inhibited the terminal differentiation of human chondrocytes in vitro and inhibits OA progression in rats in vivo [25]. PTHrP was up-regulated and mediated by calcium-sensing receptor in OA cartilage, which might promote both proliferation of chondrocyte and osteophyte formation [66]. Stimulation of focal osteochondral defect, enhancement of allograft bone union, and differentiation of MSCs are various effects of PTH (1–34) in tissue engineering [32, 67].

An ideal DMOAD can not only repair and regenerate cartilage, but also alleviate inflammation of synovium and pain. Healthy synovial joints are capable of maintaining extraordinary lubrication, attributed to structures as well as the cellular constitutions. However, both synovitis and OP contributed to cartilage degradation [68] but all pathology above could be suppressed by PTH (1–34) [69]. Impairment of SCB aggravated cartilage damage in early OPOA rabbits [39] and is associated with weight-bearing pain [70]. Overall, PTH (1–34) exhibited protective effects on the change of synovitis as well as pain relief.

Clinically, resorption played a significant role while PTH (1–34) was a reasonable option for OP patients [71]. Successful osteoanabolic treatment with PTH (1–34) benefited symptomatic stress concentration with completely stem tip pain-free [72]. The periprosthetic BMD was preserved after total hip arthroplasty (THA) [73] while bone ingrowth was promoted after total knee arthroplasty (TKA) [74] enforced by PTH (1–34). In addition, nonunion of periprosthetic fracture after TKA benefited from PTH (1–34) as well [75]. However, early mineralization of the MCC caused by PTH (1–34) might shift modifications of the subarticular spongiosa. Overall, we had better use the PTH (1–34) in proper situations and dosages.

There are some limitations in our current review. Firstly, the present review cannot identify the mechanisms accounting for the precious mechanism of PTH (1–34) on OA. Further research evidence is needed to deepen our current review. Secondly, although a thorough search was performed from five English databases, some pertinent studies may still have been missed. Thirdly, limited information in the current reviewed investigations is an urgent call for subsequent studies to confirm the findings based on additional information. Finally, there are only included studies published in English; thereby, some studies in other languages would be missed out.

## Conclusion

In conclusion, the SR, which included both in vivo and in vitro studies, described the beneficial effects of PTH (1–34) on OA via alleviating cartilage damage progression, inhibiting the abnormal SCB remodeling, suppressing synovitis, reducing oxidative stress or apoptosis of chondrocytes, and elevating autophagy. Some of the OA or OAOP patients might benefit from PTH (1–34) as well. The present SR is a description of existing studies regarding the effectiveness of PTH (1–34) administration in OA together with mechanisms, which suggested the necessity for further clinical trials and animal investigations to achieve concise conclusions about the effects of PTH (1–34) on OA.

## Supplementary Information

**Additional file 1. **Search strategy

## Data Availability

Data are available from the corresponding authors upon reasonable request with the permission of Department of Bone and Joint Surgery in Peking University Shenzhen Hospital.

## Abbreviations

PTH (1–34): Teriparatide
OA: Osteoarthritis
SR: Systematic review
SCB: Subchondral bone
ECM: Extracellular matrix
COL II: Type II collagen
AGC: Aggrecan
TNF-α: Tumor necrosis factor-α
IL-1β: Interleukin-1β
DMOADs: Disease-modifying OA drugs
OP: Osteoporosis
PRISMA: Preferred Reporting Items for Systematic Review and Meta-Analysis
PROSPERO: Prospective Register of Systematic Reviews
MeSH: Medical Subject Headings
CRIS: Checklist for Reporting In-vitro Studies
CIOA: Collagenase-induced osteoarthritis
OPOA: Osteoporotic osteoarthritis
OVX: Ovariectomized
ACLT: Anterior cruciate ligament transection
MLI: Meniscal ligamentous injury
MCC: Mandibular condylar cartilage
DMM: Destabilization of the medial meniscus
SD: Sprague–Dawley
nM: Nmol/L
PIOA: Papain-induced osteoarthritis
MSCs: Mesenchymal stem cells
M: mol/L
RA: Rheumatoid arthritis
PTHrP: Parathyroid hormone-related protein
COLX: Type X collagen
MMP13: Matrix metallopeptidase 13
ADAMTS4: ADAM Metallopeptidase With Thrombospondin Type 1 Motif 4
PTH1R: Parathyroid 1 receptor
OPG: Osteoprotegerin
RANKL: Receptor activator of NF-κB ligand
COX2: Cyclooxygenase-2
iNOS: Inducible nitric oxide synthase
MAPK: Mitogen-activated protein kinase
mTOR: Target of rapamycin
THA: Total hip arthroplasty
TKA: Total knee arthroplasty
SC: Subcutaneous injection
IA: Intra-articular
PFJOA: Patellofemoral joint osteoarthritis
pM: pmol/L
μM: μmol/L
