# Supplementary material for: Potential effects of teriparatide (PTH (1–34)) on osteoarthritis: a systematic review
Source: Arthritis Res Ther. 2023 Jan 6;25:3. doi: 10.1186/s13075-022-02981-w (PMC9817404; doi:10.1186/s13075-022-02981-w)
Supplement: Supplementary file 1 — Additional file 1. Search strategy [file 13075_2022_2981_MOESM1_ESM.docx]

**Appendix: Search strategies**

**Appendix table 1. Pubmed**

| Search number | Query | Results |
| --- | --- | --- |
| #1 | (((((((((((Osteoarthritis[MeSH Terms]) OR (Osteoarthritis[Title/Abstract])) OR (Osteoarthritides[Title/Abstract])) OR (Osteoarthrosis[Title/Abstract])) OR (Osteoarthroses[Title/Abstract])) OR (Arthritis, Degenerative[Title/Abstract])) OR (Arthritides, Degenerative[Title/Abstract])) OR (Degenerative Arthritides[Title/Abstract])) OR (Degenerative Arthritis[Title/Abstract])) OR (Arthrosis[Title/Abstract])) OR (Arthroses[Title/Abstract])) OR (Osteoarthrosis Deformans[Title/Abstract]) | 109367 |
| #2 | ((((((Teriparatide[MeSH Terms]) OR (Teriparatide[Title/Abstract])) OR (hPTH (1-34[Title/Abstract]))) OR (Human Parathyroid Hormone (1-34[Title/Abstract]))) OR (Parathar[Title/Abstract])) OR (Teriparatide Acetate[Title/Abstract])) OR (Forteo[Title/Abstract]) | 4350 |
| #1 AND #2 | ((((((((((((Osteoarthritis[MeSH Terms]) OR (Osteoarthritis[Title/Abstract])) OR (Osteoarthritides[Title/Abstract])) OR (Osteoarthrosis[Title/Abstract])) OR (Osteoarthroses[Title/Abstract])) OR (Arthritis, Degenerative[Title/Abstract])) OR (Arthritides, Degenerative[Title/Abstract])) OR (Degenerative Arthritides[Title/Abstract])) OR (Degenerative Arthritis[Title/Abstract])) OR (Arthrosis[Title/Abstract])) OR (Arthroses[Title/Abstract])) OR (Osteoarthrosis Deformans[Title/Abstract])) AND (((((((Teriparatide[MeSH Terms]) OR (Teriparatide[Title/Abstract])) OR (hPTH (1-34[Title/Abstract]))) OR (Human Parathyroid Hormone (1-34[Title/Abstract]))) OR (Parathar[Title/Abstract])) OR (Teriparatide Acetate[Title/Abstract])) OR (Forteo[Title/Abstract])) | 35 |

**Appendix table 2. Web of Science**

| Search number | Query | Results |
| --- | --- | --- |
| #1 | AB=(Osteoarthritis) OR AB=(Osteoarthritides) OR AB=(Osteoarthrosis) OR AB=(Osteoarthroses) OR AB=(Arthritis, Degenerative) OR AB=(Arthritides, Degenerative) OR AB=(Degenerative Arthritides) OR AB=(Degenerative Arthritis) OR AB=(Arthrosis) OR AB=(Noninflammatory) | 121725 |
| #2 | AB=(Teriparatide) OR AB=(hPTH (1-34)) OR AB=(Human Parathyroid Hormone (1-34)) OR AB=(Parathar) OR AB=(Teriparatide Acetate) OR AB=(Forteo) OR AB=(parathyroid hormone [1-34] peptide) | 3776 |
| #3 | #1 AND #2 | 26 |

**Appendix table** **3. Medline**

| Search number | Query | Results |
| --- | --- | --- |
| #1 | AB Osteoarthritis OR AB Osteoarthroses OR AB Osteoarthrosis OR AB Arthritis OR AB Degenerative OR AB Arthritides OR AB Degenerative OR AB Degenerative Arthritis OR AB ( Osteoarthritides OR Noninflammatory arthritis ) OR AB Degenerative Arthritides OR AB Osteoarthrosis Deformans OR AB Arthrosis | 269700 |
| #2 | AB Teriparatide OR AB parathyroid hormone[1-34] OR AB parathormone 1 34 OR AB hPTH (1-34) OR AB Human Parathyroid Hormone (1-34) OR AB Parathar OR AB Teriparatide Acetate OR AB Forteo OR AB parathyroid hormone (1-34) OR AB teriparatide recombinant human OR AB forsteo OR AB teriparatide (rDNA origin) | 2964 |
| #3 | #1 AND #2 | 90 |

**Appendix table 4. the Cochrane library**

| Search number | Query | Results |
| --- | --- | --- |
| #1 | (Osteoarthritis OR Arthritides):ti,ab,kw OR (Osteoarthroses OR Degenerative):ti,ab,kw OR (Osteoarthrosis OR Degenerative Arthritis):ti,ab,kw OR (Arthritis OR Osteoarthritides):ti,ab,kw OR (Degenerative OR Degenerative Arthritides OR Osteoarthrosis Deformans OR Arthroses OR Arthrosis):ti,ab,kw | 45088 |
| #2 | (Teriparatide):ti,ab,kw OR (Human Parathyroid Hormone):ti,ab,kw OR (hPTH):ti,ab,kw OR (Forteo):ti,ab,kw OR (Teriparatide Acetate OR Parathar):ti,ab,kw | 2617 |
| #3 | #1 AND #2 | 101 |

**Appendix table** **5. Embase**

| Search number | Query | Results |
| --- | --- | --- |
| #1 | (((((osteoarthritis:ta,ab OR arthritis:ta,ab OR degenerative) AND arthritis:ta,ab OR noninflammatory:ta,ab OR arthrosis:ta,ab OR degenerative) AND arthritis:ta,ab OR degenerative) AND ('joint'/exp OR joint) AND disease:ta,ab OR noninflammatory) AND arthritis:ta,ab OR osteoarthritis:ta,ab OR osteoarthrosis:ta,ab OR primary) AND osteoarthritis:ta,ab | 102435 |
| #2 | (((('parathyroid'/exp OR parathyroid) AND 'hormone1 34':ta,ab OR 'parathormone'/exp OR parathormone) AND 1 AND 34:ta,ab OR 'parathormone'/exp OR parathormone) AND '1 34':ta,ab OR 'parathyroid'/exp OR parathyroid) AND ('hormone'/exp OR hormone) AND '1 34' AND peptide:ta,ab OR teriparatide:ta,ab OR forsteo:ta,ab OR parathar:ta,ab | 4378 |
| #3 | #1 AND #2 | 44 |
